# A Targeted Association Study of Immunity Genes and Networks Suggests Novel Associations with Placental Malaria Infection

**DOI:** 10.1371/journal.pone.0024996

**Published:** 2011-09-19

**Authors:** Martin Sikora, Hafid Laayouni, Clara Menendez, Alfredo Mayor, Azucena Bardaji, Betuel Sigauque, Mihai G. Netea, Ferran Casals, Jaume Bertranpetit

**Affiliations:** 1 Institute of Evolutionary Biology (UPF-CSIC), CEXS – UPF – PRBB, Barcelona, Catalonia, Spain; 2 CIBER en Epidemiología y Salud Pública (CIBERESP), Madrid, Spain; 3 Barcelona Center for International Health Research (CRESIB), Hospital Clinic, Institut d'Investigacions Biomedicas August Pi i Sunyer (IDIBAPS), Universitat de Barcelona,Barcelona, Spain; 4 The Manhiça Health Research Center (CISM), Manhiça, Mozambique; 5 Department of Medicine, Radboud University Nijmegen Medical Center, Nijmegen, The Netherlands; Instituto Gulbenkian de Ciência, Portugal

## Abstract

A large proportion of the death toll associated with malaria is a consequence of malaria infection during pregnancy, causing up to 200,000 infant deaths annually. We previously published the first extensive genetic association study of placental malaria infection, and here we extend this analysis considerably, investigating genetic variation in over 9,000 SNPs in more than 1,000 genes involved in immunity and inflammation for their involvement in susceptibility to placental malaria infection. We applied a new approach incorporating results from both single gene analysis as well as gene-gene interactions on a protein-protein interaction network. We found suggestive associations of variants in the gene *KLRK1* in the single gene analysis, as well as evidence for associations of multiple members of the IL-7/IL-7R signalling cascade in the combined analysis. To our knowledge, this is the first large-scale genetic study on placental malaria infection to date, opening the door for follow-up studies trying to elucidate the genetic basis of this neglected form of malaria.

## Introduction

Recent years have seen a substantial increase in efforts and funding directed at the control [1], [2] and eventual eradication of malaria [3]. However, despite these efforts, it remains one of the deadliest diseases worldwide. The global death toll has been estimated at 700,000 to 1,000,000 in 2008 alone, from a total number of cases ranging from 208 million to 276 million [4]. The majority of these deaths occur in Sub-Saharan Africa, as a consequence of infection with *Plasmodium falciparum*. A somewhat lesser known aspect of this statistics is the fact that a large fraction of this annual death toll is a consequence of malaria infection during pregnancy, with up to 200,000 deaths attributed to it [5], [6]. Women in their first pregnancy are particularly at risk of infection [7], [8], and both mothers and their offspring face a number of potential life-threatening complications as a consequence of infection [9], [10]. The key characteristic of the pathogenesis of this type of malaria is infection of the placenta, i.e. the sequestration and subsequent accumulation of infected erythrocytes in the intervillous space [11].

Genetic studies of malaria susceptibility have a long history, and a variety of host genetic factors have been implicated, most prominently the protective effect of the hemoglobin S (HbS) variant [12]. Notably, even this strong effect has been estimated to account for only roughly 2% of the total variation, indicating that a large proportion of genetic resistance factors remain to be discovered [13]. Efforts in discovering these factors have culminated in the recent publication of the first genome-wide association study (GWAS) of malaria, carried out in West Africa [14]. This study highlighted both the potential and the possible pitfalls of large-scale genetic association studies using single nucleotide polymorphisms (SNP) genotyping in African populations. However, its focus was on severe malaria, a complex compound phenotype consisting of severe anemia or cerebral malaria that occurs mostly in children under the age of five, which greatly differ in its physiopathology from malaria in pregnancy. Important differences between the pathophysiology of placental malaria and malaria occurring in non-pregnant patients have been extensively documented, both in terms of the erythrocyte surface antigens sequestered in the placenta that adhere specifically to chondroitin sulphate A, and the predominantly monocytic infiltrate present in placental malaria (for review see [11]). We therefore focused on a more easily measurable phenotype related to placental malaria, namely the susceptibility to infection of the placenta. We previously published the first extensive genetic association study of this phenotype, analyzing genetic variation in 64 genes, and reporting a significant association in the gene *FUT9* [15]. Here, we extend this analysis considerably, by investigating genetic variation in more than 1,000 genes involved in immunity and inflammation for their involvement in placental malaria infection. To our knowledge, this is the first large-scale genetic study on this particular malaria phenotype to date.

## Results

### Genotyping quality and population structure

Out of a total of 347 samples for which genotyping was attempted, 68 did not pass the quality control thresholds for cluster genotyping (see Methods) and were removed. The remaining 279 samples were assayed for a total of 9,178 SNPs. Out of those, we removed all SNPs with ambiguous mapping in the latest SNP annotation (56; Affymetrix annotation release 1.5), with a call rate below 0.8 (439), all that failed testing for Hardy-Weinberg equilibrium in controls (115; p≤10^−4^), as well as all monomorphic SNPs (1,554). This resulted in a final analysis sample of 279 individuals (173 cases, 174 controls) and 7,442 SNPs, with an average call rate of 0.97. Testing for population stratification using EIGENSOFT revealed no significant differentiation between cases and controls (p = 0.16), therefore this final sample was used without correcting for stratification in all following analyses.

### Association analysis

We performed extensive analyses to test the hypothesis that common variation in the set of host immunity and inflammatory genes is affecting the susceptibility to placental infection (defined as the presence or absence of parasites in placental tissue; see Methods).

**Figure 1:**
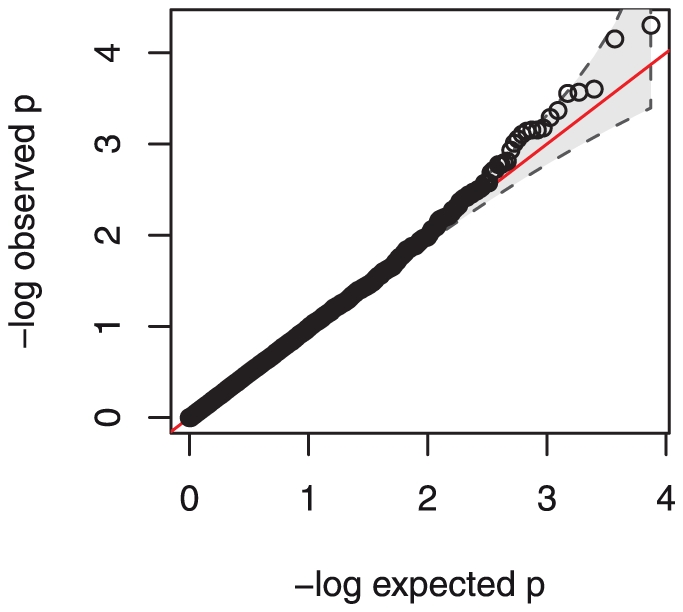
QQ plot of single SNP association statistics. The negative logarithm of the ordered empirical p-values obtained from the single marker association analysis is plotted against the negative logarithm of the ordered p-values from a uniform distribution, as expected under the null hypothesis. The grey shaded area indicates the 95% concentration band. No systematic inflation was observed in the test statistics, as noted by the majority of points falling on the diagonal (red line, y = x).

**Figure 2:**
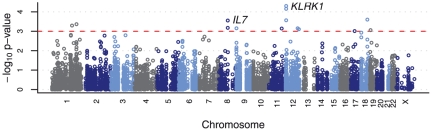
Genome-wide distribution of single SNP association statistics. Manhattan plot showing the distribution of the single SNP association statistics. Colored circles indicate the negative logarithm of the empirical p-value for all QC+ SNPs, with colors differentiating the different chromosomes. The red dashed line indicates a significance level of 10^−3^. The two candidate regions *IL7* and *KLRK1* are also indicated.

As a first step we performed single marker analysis for all SNPs, testing for five models of penetrance (see Methods). Figure 1 shows a quantile-quantile (QQ) plot of the results, confirming the absence of a bias in the distribution of p-values due to population structure, as expected from the results of the EIGENSOFT analysis. The genomic distribution of the results of the single marker association tests is shown in Figure 2. The strongest signal was seen in three neighboring SNPs in the region of the gene *KLRK1* on chromosome 12 (see Table 1), with the best SNP showing p = 5×10^−5^ (rs12821887). However, none of the SNPs reached genome-wide significance after correcting for multiple testing. In the absence of a clear association signal, we chose to further investigate all regions showing multiple SNPs among the top signals. The *KLRK1* region contains three of the top four among all tested SNPs within a region of 8 kb, and therefore merits a more thorough investigation. Another region of potential interest was found in the gene *IL7* on chromosome 8, where two neighboring SNPs 33 kb apart were also found among the top genome wide 10 association signals. Results for those two genes remain significant after inclusion of age, parity as well as peripheral malaria infection as covariates in a logistic regression analysis (determined from blood drawn at a visit pre-delivery) (Table 2).

**Table 1:** Top 10 single SNP association signals.

SNP	Chr^a^	Position	Alleles^b^	Best test^c^	Affected	Unaffected	df^d^	p_test_ e	p^f^	p^g^	Gene
rs12821887	12	10,445,946	C/T	D	47/92	82/57	1	2.56E-05	5.00E-05	0.21	*KLRK1*
rs728010	12	10,444,534	A/G	D	47/91	80/58	1	6.74E-05	7.00E-05	0.47	*KLRK1*
rs2850760	18	59,117,803	C/T	A	35/243	11/269	1	2.00E-04	2.50E-04	0.85	*BCL2*
rs7972757	12	10,437,407	G/A	A	36/220	75/191	1	7.98E-05	2.70E-04	0.53	*KLRK1*
rs2583764	8	79,826,355	A/G	A	128/144	85/189	1	1.22E-04	2.80E-04	0.69	*IL7*
rs1571344	1	205,737,551	G/A	R	16/121	41/94	1	1.53E-04	4.30E-04	0.76	*CR1*
rs3917422*	1	167,965,384	G/T	T	15/261	2/278	1	1.02E-03	5.10E-04	1.00	*SELE*
rs2583762	8	79,860,038	A/T	T	128/144	90/190	1	3.03E-04	6.70E-04	0.96	*IL7*
rs3824433	9	5,103,577	C/T	D	59/71	84/39	1	2.39E-04	7.00E-04	0.89	*JAK2*
rs7486905	12	105,684,299	G/A	R	12/104	35/86	1	3.36E-04	7.00E-04	0.96	*RFX4*

a Chromosome.

b minor/major allele (positive strand).

c test model with lowest asymptotic p-value: D, dominant; A, allelic; R, recessive; T, trend.

d degrees of freedom.

e p_test_, asymptotic p-value from best test model.

f p_nominal;perm_, empirical p-value for respective SNP, overall for all tested models; 100,000 permutations.

g p_corrected;perm_; empirical p-value for respective SNP, corrected for all 7442 tested SNPs.

*rs3917422 is a non-synonymous coding SNP (Pro / Gln).

All SNPs are within the gene region, except the last at 3588 bp.

**Table 2:** Logistic regression results for top SNPs in *KLRK1* and *IL7*.

SNP	Model^a^	Gene	P unadjusted	P parity	P parity, age, peripheral infections
rs12821887	Dominant	*KLRK1*	3.16E-05	2.46E-05	6.09E-05
rs728010	Dominant	*KLRK1*	7.99E-05	5.60E-05	1.16E-04
rs7972757	Allelic	*KLRK1*	1.04E-04	2.25E-04	6.46E-04
rs2583764	Allelic	*IL7*	1.34E-04	1.02E-04	1.66E-04
rs2583762	Trend	*IL7*	3.85E-04	3.75E-04	5.13E-04

a Models tested were the ones with the strongest association in the genome-wide analysis (Table 1).

**Figure 3:**
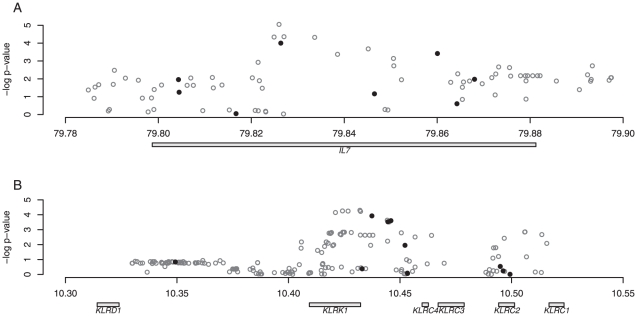
Imputation association results at *IL7* and *KLRK1*. Results of the association analysis using imputation in the two candidate regions *IL7* (A) and *KLRK1* (B). In both regions, the negative logarithm of the empirical p-values is plotted (see Methods for details), with filled circles indicating the SNPs genotyped in the respective regions, and empty circles indicating imputed SNPs using the full HapMap 2 panel as a reference. Chromosomal positions of the genes in the region are indicated below the respective panel.

In order to get a more fine scale resolution of the results in both of those regions, we used BIMBAM to impute genotypes at untyped SNPs and test them for association, using the combined HapMap 2 as a reference panel. We chose to use this reference panel following the suggestions of Guan and Stephens [16], who found that accuracy was improved for situations where the population analyzed was not well represented by any single reference panel. Nevertheless, results from this analysis have to be interpreted with care, as even the African population in the reference panel (Yorubans from Nigeria) shows considerable geographic as well as genetic distance from the study population from southern Mozambique [17]. Figure 3 shows the result of the analysis. As expected by the much higher density of the reference panel, both regions show imputed SNPs with stronger association signals than any of the genotyped SNPs. Around rs2583764, the top signal of the genotyped SNPs in the *IL7* region, a number of imputed SNPs with lower p-values are found. The top SNP in this region (rs2583763, p = 9×10^−6^), 392 bp upstream of rs2583764, is also the top hit among all imputed SNPs. In the *KLRK1* region, a number of imputed SNPs within the gene region also show stronger evidence (top SNP rs7962112, p = 5×10^−5^).

Finally, in order to investigate whether power to detect association in our sample could be improved by using combinations of multiple markers, we carried out haplotype association tests using BEAGLE. The strongest signal in this analysis was found in the gene *IL7*, where a cluster including rs2583764 was close to the significance level after a strict multiple test correction (minimum p = 3.2×10^−6^; permutation p = 0.07). Dissecting this cluster association result further, we found that a haplotype of four SNPs (ATGA; rs1441438 - rs1036751 - rs6993386 - rs2583764) explained the observed signal. A standard association test for the ATGA haplotype against all other observed haplotypes at the locus indicated a susceptibility effect (OR_ATGA_ = 3.1; 95% CI = 1.9–5.0; p = 1.6×10^−6^). Not surprisingly, this 22 kb haplotype also spans the SNP rs2583763 found in the imputation analysis.

### Gene-gene interactions and network analysis

Given that our study focuses on genes related to specific organismal functions, namely inflammation and immunity, we next set out the analysis to include information on the interactions of those genes and their organization in cellular pathways in our analysis. We therefore performed SNP×SNP interaction association tests in our sample. In order to reduce the number of tests, we only analyzed those interacting SNP pairs that were found within 5 kb of genes that had evidence for interaction of their respective protein products. To that end, curated protein interactions were obtained from the Michigan Molecular Interactions database (see Methods). Querying the database with the genes included in the study resulted in a network consisting of a total of 892 genes (nodes), connected by 3789 interactions (edges), from now on referred to as “immunity network”. We then tested all resulting SNP pairs for interaction effects using logistic regression. The strongest signal comes from a SNP pair located in the genes *IL7R* (rs1494558) and *JAK3* (rs6512227). However, this was again not significant after correcting for multiple testing (nominal p = 5.4×10^−5^), but given the still considerable number of tests in this pair wise analysis, this is not unexpected.

Even so, two interesting properties of this SNP pair called our attention: firstly, one of the two SNPs, rs1494558, is a non-synonymous variant responsible for a non-polar to polar amino acid substitution (Ile→Thr; Grantham distance D = 89 [18]); and second, *IL7R* is also interacting with *IL7*, one of the two regions found with the strongest signal in the single marker analysis. We therefore investigated the joint distribution of association results for both genes and interactions on the immunity network in more detail, using two different approaches.

**Figure 4:**
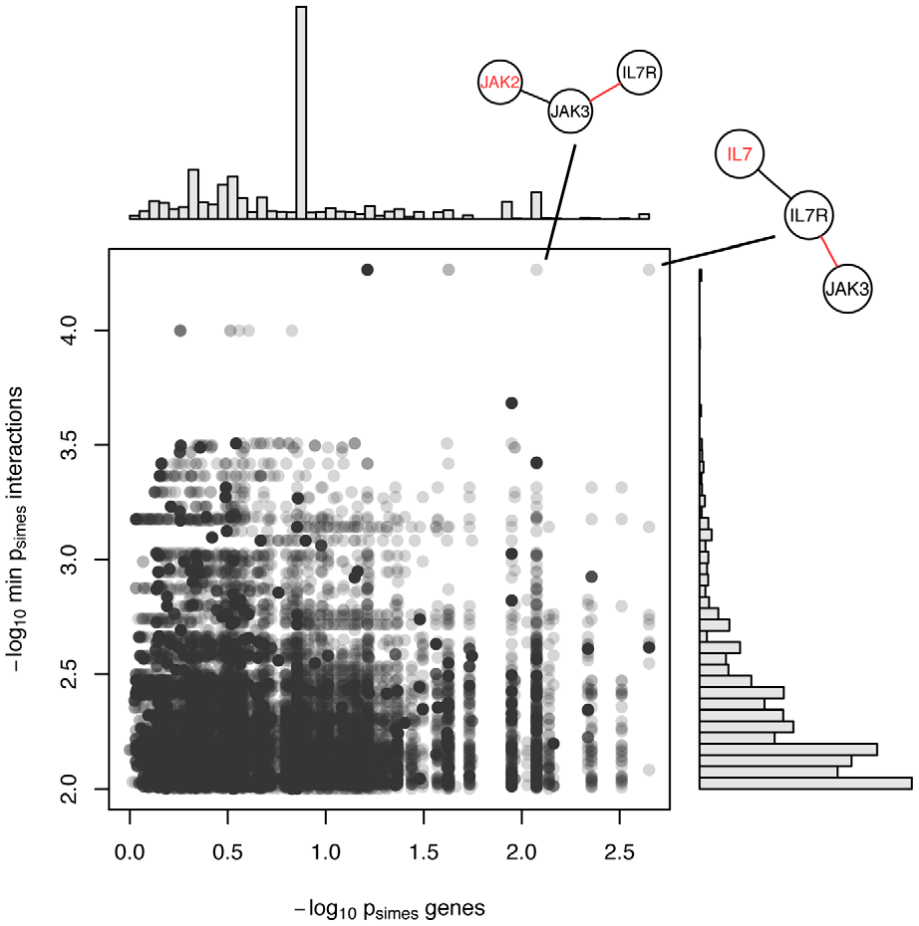
Distribution of gene and interaction p-values for chain motifs. Plot showing the distribution of minimum gene p-value versus minimum interaction p-value for each chain motif of length three, on a negative logarithmic scale. For clarity, only interactions with p< = 10^−2^ are shown. In order to deal with the considerable amount of overplotting due to the large number of data points with similar values, alpha transparency is used for color, resulting in darker colors in regions with many overlapping points. Histograms show the marginal distributions of the minimum gene / interactions p-values, respectively. The structure of the two outlier motifs containing the interaction *IL7R* – *JAK3* is also depicted. The gene / interaction corresponding to the values on the plot are indicated in red.

**Figure 5:**
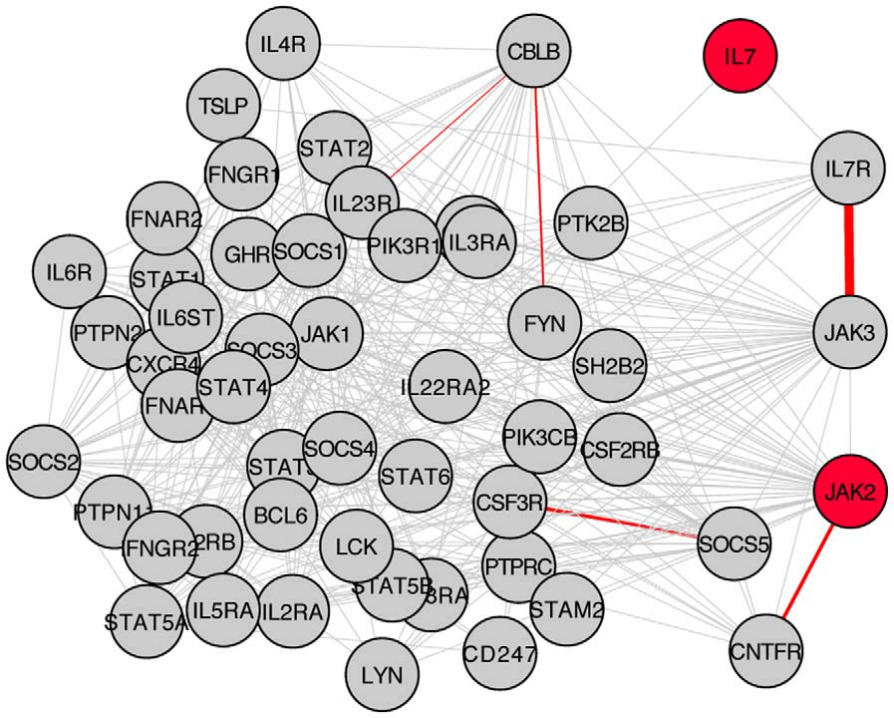
Sub-network of first neighbours of *IL7R* – *JAK3* interaction. A sub-network of the full immune network, containing all nodes separated by one edge from the *IL7R* – *JAK3* interaction (52), and all their interactions (425). Nodes in red indicate genes with p<10^−2^. Edges in red indicate interactions with p<10^−3^, with thicker lines corresponding to lower p-values. The *IL7* module is shown on the right part of the plot.

In the first approach, we assigned a single p-value to each gene and interaction, by combining all SNP p-values mapped to a particular gene or interaction using the Simes method (see Material and Methods). We chose this method over a simpler minimum p-value method in order to avoid systematic biases due to differing number of SNPs in the respective genes/interactions. Having obtained the gene- and interaction-wise p-values, we wanted to see how unusual it was to find a low interaction p-value at one edge distance to a low gene p-value in the immunity network. To this end, we employed a network motif search algorithm to first identify all distinct network motifs composed of three nodes connected by two edges (i.e. chain motifs of length three) in the network. After having obtained the list of motifs (116,728 total), we plotted the distribution of the minimum p-values of the genes versus the minimum p-values of the interaction, for each of the motifs. Results are shown in Figure 4. As can be seen in the distribution, the motif containing both *IL7* and the neighboring interaction *IL7R* – *JAK3* is a clear outlier in the empirical distribution. Furthermore, the only other point that also behaves as an outlier for both genes and interactions is an overlapping motif to the former one, including the same *IL7R* - *JAK3* interaction together with *JAK2*, a gene downstream of *JAK3*. A more detailed look at the subnetwork containing all first neighbors of the *IL7R* - *JAK3* interaction is shown in Figure 5. As can be seen, the strongest signals in both genes and interactions are clustered in the module *IL7-IL7R-JAK3-JAK2-CNTFR*, which is part of the *IL7* signaling cascade.

**Figure 6:**
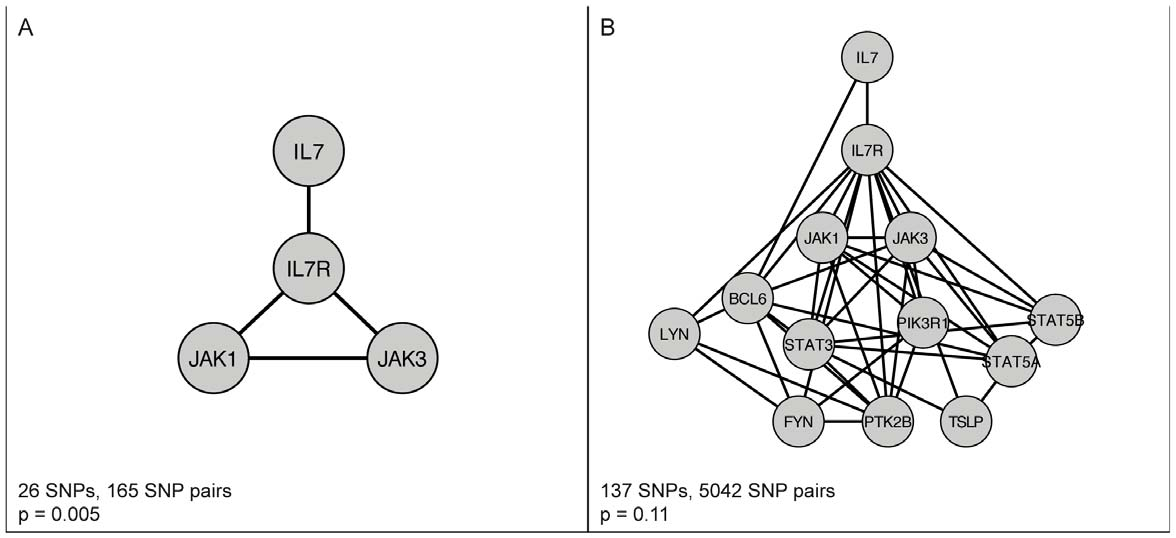
ARTP of *IL7* sub-networks. Sub-networks used for the ARTP analysis and their results. (A) *IL7* “core” module; (B) extended module considering all first neighbor interactions of *IL7R*.

Given the results of this first approach, we wanted to investigate the overall statistical evidence for association of the *IL7* signaling cascade in more detail. To this end, we performed an adaptive combination of p-values as described in Yu et al. [19], a flexible method that allows the combination of evidence across multiple SNPs and/or genes in a pathway or network (see Methods for details). Since we were interested in combining evidence both for single SNP factors as well as SNP×SNP interactions, we computed p-values through logistic regression analysis of each SNP pair in the network of interest, comparing a full model including main effects of both SNPs and their interaction effect against a baseline model without any SNP effects. As in the previous analysis, we considered only SNP pairs that were found within 5 kb of genes that had evidence for interaction of their respective protein products, thereby taking into account the topology of the network of interest. The list of p-values was then subjected to the ARTP procedure to obtain an overall measure of significance. We performed this analysis for two different sub-networks: The “core” module of *IL7* signaling comprised of *IL7*, its receptor and the immediate downstream adaptor molecules *JAK1* and *JAK3*; and a more extended module considering all first neighbors of the receptor *IL7R* in the network (Figure 6).

Results of this analysis suggest a strong overall association of the *IL7* core module (p = 0.005; 1000 permutations), whereas the larger extended module does not reach significance (p = 0.11). Taken together, these results indicate a potential role for this signal-transduction module in the susceptibility to placental malaria infection.

## Discussion

The present study is the first large-scale survey on human genetic variation in the immune system and inflammatory response and its relationship to malaria in pregnancy. It is clear that both of these cellular processes are important components in the response to malaria infection, making them strong candidates for harboring loci that influence susceptibility. Nonetheless, our results show no single region standing out with a clear signal of association, if we consider the stringent statistical thresholds normally employed for GWAS. Explanations for the lack of a clear candidate can be manifold, from a genuine lack of susceptibility loci in the analyzed genes to a lack of power to detect loci with weak effects due to small sample size. For example, we note that the focus of our study was on a targeted subset of the human genome, namely a set of genes related to inflammation and immunity. It is clearly a possibility that other regions not covered by our study could harbor variants that have a strong effect on placental infection either by themselves or in interaction with the genes in our targeted set. In this context, the logical next step would be a well-powered GWAS on thousands of samples with an adequate high density SNP array. Unfortunately, the major limitation in our case was the number of well-phenotyped samples available, making this approach unfeasible. It is however also important to point out that our results mirror the observations of Jallow *et al.* [14] in their malaria GWAS in West Africa, namely the difficulty of achieving the thresholds normally applied in studies with samples of European descent in Africa. In their study, even in the *HBB* region, with its known strong protective effect due to hemoglobin S, the strongest signal only barely passes the normally applied threshold of p = 5×10^−7^ (p = 3.9×10^−7^) [20]. The reason is that due to generally lower levels of linkage disequilibrium (LD) in Africa and genotyping arrays being designed using tagSNPs derived from European populations, effective coverage of even common variants in African populations is low. The targeted genotyping array used in our study will evidently suffer from the same limitations. We therefore followed their example and considered loci with p<10^−4^ as interesting regions. The only region that achieves that threshold is *KLRK1*, which codes for a C-type lectin receptor expressed on the surface of T-cells and natural killer cells [21]. Its role lies in activation of those cells as a response to viral infections or certain tumors. It is therefore not immediately clear how it could exert a potential effect on placental malaria infection. While it is certainly a possibility that there is an effect via an unanticipated mechanism, this result has to be taken with caution until confirmed through a replication study. In addition to that, *IL7* emerged as another potential candidate locus, based on the results of both imputation and haplotype association, as well as in the gene-gene interaction analysis, which will be discussed below.

An important limitation of our study is that due to our study design, we cannot formally rule out the possibility that the observed associations are with malaria infection overall, and not specific to placental infection. For example, if placental malaria infection would be associated with high levels of parasitemia, and a variant is associated with parasitemia, we would also expect to observe an association of the variant with placental malaria infection. In order to formally rule out this possibility, it would have been necessary to test for placental infection conditional on an observed peripheral infection. Although peripheral malaria status (determined from blood drawn at a visit pre-delivery) was available as a phenotype, in total there were only 39 individuals that showed evidence of acute malaria infection, and only two of those were control individuals (i.e. without placental infection), making the suggested study design unfeasible. However, performing logistic regression on the top SNPs, adjusting for peripheral malaria infection status as confounder together with parity and age does not change the strength of the association of the SNPs significantly, which would argue against this possibility. Additionally, we also confirmed that although primigravid women are significantly more frequent among the cases (p = 0.006, Fisher's exact test), the top SNPs remain significant after controlling for parity as potential confounder.

A more general concern with association studies is the focus on single genes, without taking into account the interactions among them and their organization into functional pathways. Recent effort has therefore been aimed at incorporating this knowledge into the analysis, both in the development of methods for detecting gene-gene interactions (reviewed in [22]) as well as in the analysis of genetic association studies in the context of biological networks [23], [24], [25]. In this study, we performed an in-depth analysis to integrate the results of both single gene and gene – gene interaction tests in the context of the known interactions of the immunity network. Results of this analysis give additional support to a role of IL-7 signaling in modulating susceptibility to placental infection. Furthermore, results suggest that this role is restricted to the core of the IL-7 signaling module (Figure 6A), composed only of IL-7 and its receptor, as well as its immediate adaptor molecules. The signal is lost when considering a more extended network of downstream components (Figure 6B), due to the noise introduced from the larger number of genes without evidence for association. Integration of interaction data can therefore overcome some of the problems mentioned above, and can aid in prioritizing candidates in the absence of clear association signals. Nonetheless, there is still a general lack of powerful statistical tools to disentangle the effects of multiple interacting variants at different loci on a phenotype of interest, which, when becoming available, will certainly be of great impact in the mapping of genotypes to phenotypes.

Based on these results we therefore suggest a role of IL-7/IL-7R signaling in susceptibility of placental malaria infection. The module identified forms part of the JAK-STAT signaling pathway, which regulates cellular responses mediated by binding of cytokines like IL-7. Some of the responses mediated upon binding of cytokines include cell proliferation and differentiation, making it a key pathway in processes like hematopoiesis and immune development [26]. IL-7 in particular is an important factor in B and T cell development. Looking in more detail at the interaction results, it was intriguing that the top result involved an interaction with a non-synonymous SNP in *IL7R*. The variant (rs1494558) causes a change from Threonin to Isoleucin in the extracellular domain of the receptor. It is, together with other variants, implicated in autosomal recessive severe combined immunodeficiency, although impairment of IL-7 signaling was not observed [27]. The observed effect is a dominant interaction with rs6512227 (Figure S1), a SNP located upstream of *JAK3*, indicating a potential regulatory effect. The gene codes for the tyrosine kinase JAK3, an intracellular adaptor protein that is involved in the transduction of signal induced by cytokine receptor binding.

The involvement of the IL-7/IL-17R pathway in placental malaria may be relatively unexpected, but not illogical. Although the most studied activities of IL-7 are those related to B- and T-cell proliferation, IL-7 also exerts important proinflammatory effects. IL-7 has been shown to induce production of TNF by T- and B-cells [28], an important proinflammatory cytokine with deleterious effects in placental malaria. Moreover, IL-7 has been described to drive inflammation in several prototypic inflammatory conditions such as rheumatoid arthritis [29] or atherosclerosis [30]. The inflammatory properties of IL-7/IL-7R pathway could influence susceptibility to placental malaria infection by acting at two separate levels: by modulating direct antimalarial immunity and resistance to infection, and by modulating the inflammatory reaction in the placenta during infection, with subsequent consequences for the outcome of the pregnancy. Since we had data on the pregnancy outcome of the study individuals available, we investigated this possibility by testing the association of pregnancy outcomes (premature delivery and birth weight) with the top SNPs in *IL7*, as well as the significant SNP×SNP interaction. However, none of those were significant.

In conclusion, our results show evidence for association with placental malaria infection at *KLRK1*, a gene encoding a natural killer cell receptor molecule and point towards a likely role for IL-7 signaling through IL7R and the JAK/STAT intracellular adaptors. Our study is the first large-scale attempt to determine the genetic basis of placental infection in malaria, and suggests an important unexpected role of the IL-7/IL-7R pathway for the susceptibility of this important clinical condition.

## Materials and Methods

### Ethics Statement

The study received ethical clearance by the National Mozambican Ethics Review Committee and the Hospital Clinic of Barcelona Ethics Review Committee.

### Study subjects

The subjects enrolled in this study have been described in detail elsewhere [15], [31]. Briefly, 360 pregnant women from Manhiça District, southern Mozambique, were chosen among the placebo group of a malaria control intervention trial to form a nested case-control sample of 180 cases and 180 controls. Our phenotype of interest was placental malaria infection, which was defined as the presence of asexual *Plasmodium falciparum* parasites and/or malaria pigment in placental tissue samples. Cases were chosen as pregnant women with evidence of acute, past, or chronic placental malaria infection, whereas controls were pregnant women who did not show any evidence of parasite infections in the placenta. We chose cases and controls solely on the basis of infection status of the placenta, irrespective of actually observed acute malaria infection of the individuals.

### Genotyping

Genotyping was performed using the Affymetrix GeneChip Human Immune and Inflammation 9K SNP Kit, which contains approximately 9,000 SNPs located in around 1,000 genes related to the human immune and inflammatory response. Sample preparation before genotyping consisted in DNA extraction from dried blood spots on filter paper, followed by whole genome amplification (GenomiPhi kit, GE Healthcare). Genotype calling was performed using the Affymetrix GeneChip Targeted Genotyping Analysis Software (Version 1.6). Samples were included for cluster genotyping according to the following criteria: QC call rate>75%; QC half rate<17%; Signal/background ratio>20. Cluster genotyping was performed using the standard settings with the exception of parameter “MinCallConfidence”, which was reduced to 0.8 in order to increase the number of SNPs with raw genotype calls (before applying quality control data filtering, as described in the results).

### Statistical analysis

Genotype data management and filtering, as well as single marker association tests and SNP-SNP interaction tests were carried out using PLINK (version 1.06) [32]. Five tests of association were applied for each SNP: allelic, dominant, recessive, full genotypic, and the Cochran-Armitage trend test. Both pointwise and multiple testing corrected estimates of empirical p-values were obtained by permutation as implemented in PLINK (100,000 replicates). SNP-SNP interaction tests were performed for all pairwise combinations, using the logistic regression option in PLINK.

Genotype imputation and Bayesian association mapping for candidate regions were performed using BIMBAM (version 0.99) [33]. Due to the lack of an appropriate Southeast African reference panel, we used the combined panel of all three HapMap populations as a reference for imputation to minimize error rates [16]. All SNPs within 20 kb of the candidate regions were considered for imputation. Bayes factors were transformed to p-values using permutation (1,000,000 replicates) [34].

Multi-marker association analysis was performed using BEAGLE (version 3.0.2) [35], [36], using the allelic, dominant and recessive tests. In order to account for the expected greater haplotypic diversity and therefore increased number of distinct haplotype clusters in African samples, we reduced the ‘scale’ parameter to 2. Empirical p-values were obtained using permutation (10,000 replicates). Phase estimation prior to analysis was carried out using fastPHASE (version 1.4.0) [37] with default settings.

Protein interaction network visualization and analysis was performed using Cytoscape (version 2.6.3) [38], as well as R (version 2.9.0) and Bioconductor (version 2.4) [39]. Protein interaction data was obtained using the Michigan Molecular Interactions (MiMI) plugin for Cytoscape (version 3.0.1) [40]. Network motif search was carried out using the tYNA web tool [41]. SNP-SNP interaction association statistics were mapped to a particular protein-protein interaction if both SNPs were found within 5 kb of the respective genes.

Gene-wise and interaction-wise p-values were obtained by combining the p-values of all SNPs within 5 kb of a gene, or of all SNP-SNP interactions mapped to a particular protein-protein interaction, respectively. P-values were combined using the Simes procedure:
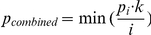
where p_i_ is the i^th^ ordered p-value of all k SNPs mapped to a particular gene or interaction. This was chosen instead of a simpler minimum p-value method, in order to avoid bias towards lower combined p-values in genes/interactions with larger number of SNPs.

The adaptive rank truncated product (ARTP) procedure for p-value combination was performed as in Yu et al. [19], and is briefly described here. The method uses a permutation procedure to obtain an empirical overall significance from a list of observed p-values. We therefore generated 1000 permuted datasets under the null hypothesis of no association, by randomly reassigning case and control labels of the samples using PLINK. Next, p-values for the ARTP were obtained by logistic regression analysis of each SNP pair in the network of interest, comparing a full model including main effects of both SNPs and their interaction effect against a baseline model without any SNP effects, both for the observed and each of the 1000 permuted datasets. Each of those vectors of p-values was then ordered, and the product of the K smallest p-values was calculated for each, using different values for the truncation point K. The truncation points we used were K = 1, 2, 5, 10, 15, and 20. For each of the truncation points (i.e. rows) in the resulting matrix of 1001 datasets×6 values of K, a p-value for both the observed as well as the 1000 permuted datasets was calculated from the full distribution of the p-value products of that particular K. The final adjusted p-value for the ARTP of the observed data was then obtained by comparing its minimal p-value over all values of K against the full distribution of minimal p-values for each dataset over all values of K.

Population structure was analyzed using EIGENSOFT (version 2.0) [42], with default settings and correcting for linkage disequilibrium (LD). Population differentiation (*F_ST_*) and selection statistics (iHS) of the Illumina 650k Human Genome Diversity Panel (HGDP) data [43] as well as HapMap data [44] were calculated as previously described [15]. Data visualization and additional statistical analysis was performed using R.

## Supporting Information

Figure S1 **Interaction effect of rs1494558–rs6512227 interaction.** Log odds of disease for all allelic combinations of the two SNPs, estimated by logistic regression.
(TIFF)
