# Supplementary figures and images for: A Targeted Association Study of Immunity Genes and Networks Suggests Novel Associations with Placental Malaria Infection
Source: PLoS One. 2011 Sep 19;6(9):e24996. doi: 10.1371/journal.pone.0024996 (PMC3176307; doi:10.1371/journal.pone.0024996)

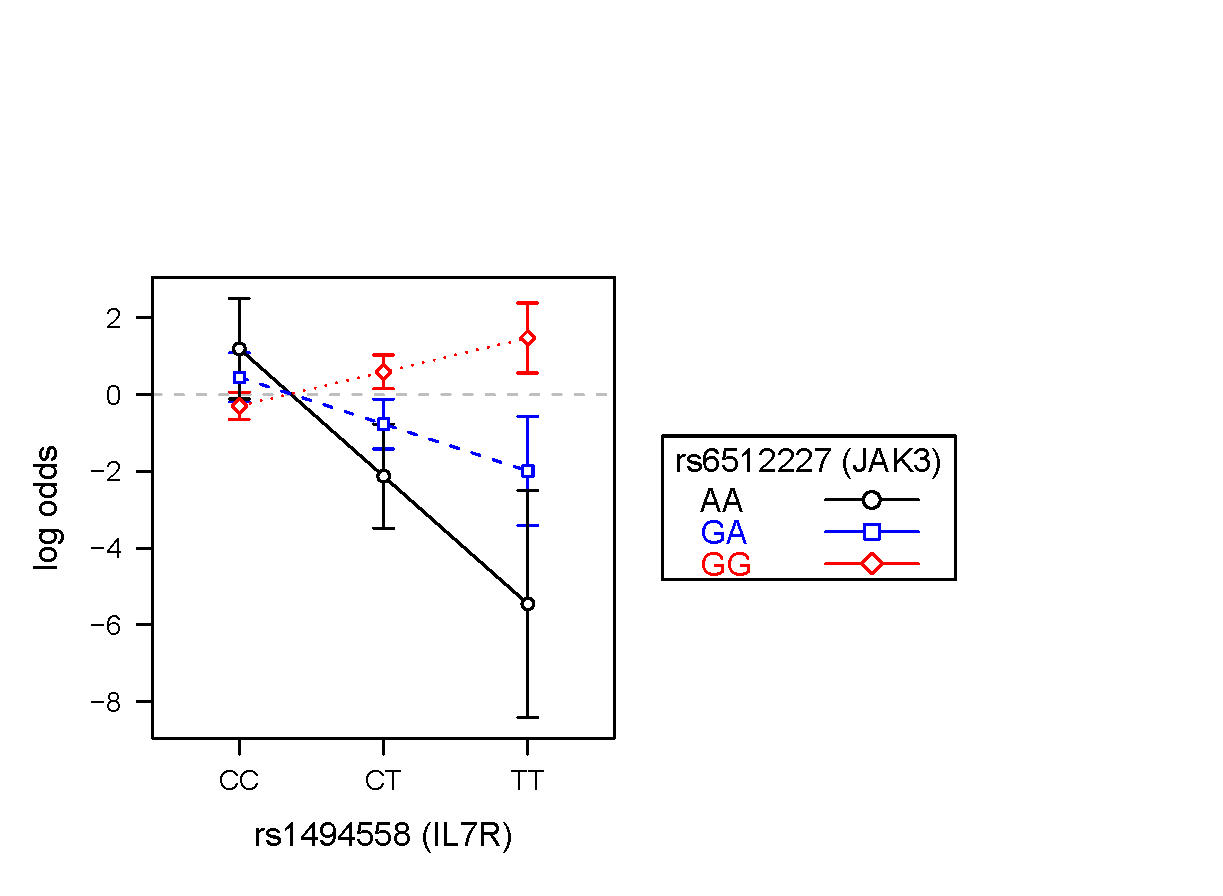

Supplement: Figure S1 — Interaction effect of rs1494558–rs6512227 interaction. Log odds of disease for all allelic combinations of the two SNPs, estimated by logistic regression. (TIFF) [file pone.0024996.s001.tiff]
